# Prevalence of Sjögren’s syndrome associated with rheumatoid arthritis in the USA: an observational study from the Corrona registry

**DOI:** 10.1007/s10067-020-05004-8

**Published:** 2020-03-04

**Authors:** Leslie R. Harrold, Ying Shan, Sabrina Rebello, Neil Kramer, Sean E. Connolly, Evo Alemao, Sheila Kelly, Joel M. Kremer, Elliot D. Rosenstein

**Affiliations:** 1 Corrona, LLC, 1440 Main Street, Suite 310, Waltham, MA 02451 USA; 2 University of Massachusetts Medical School, Worcester, MA USA; 3 Institute for Rheumatic & Autoimmune Diseases, Overlook Medical Center, Summit, NJ USA; 4 Bristol-Myers Squibb, Princeton, NJ USA; 5 Albany Medical College and the Center for Rheumatology, Albany, NY USA

**Keywords:** Observational study, Prevalence, Rheumatoid arthritis, Sjögren’s syndrome

## Abstract

The objectives of this analysis were to assess the prevalence of Sjögren’s syndrome (SS) associated with rheumatoid arthritis (RA) and to compare baseline characteristics of patients with RA with and without SS. Adult patients with RA from a large observational US registry (Corrona RA), with ≥ 1 visit for assessment of SS status between 22 April 2010 and 28 February 2018, were considered. Patients with RA with versus without SS were compared. SS status was determined from a yes/no variable and reported at enrollment into the Corrona RA registry and follow-up visits. Outcomes were unadjusted prevalence of SS in patients with RA, prevalence of SS by RA disease duration, and baseline characteristics in patients with RA by SS status. Of 24,528 eligible patients, 7870 (32.1%) had a diagnosis of RA and SS. The unadjusted overall rate for SS prevalence in patients with RA was 0.30 (95% confidence interval 0.29, 0.31). SS prevalence increased with increasing RA duration. Patients with RA with versus without SS were more likely to be older, female, and seropositive; had a longer RA duration; higher disease activity; and a higher incidence of comorbidities (hypertension, cardiovascular disease, malignancies, and serious infections), erosive disease, and subcutaneous nodules at index date. Patients with RA and SS had a higher disease burden than those with RA only. The prevalence of SS increased as duration of RA increased. RA with SS was associated with seropositivity, more severe RA, extra-articular manifestations, and comorbidities.

**Table:** 

**Key Points** • *The overall prevalence of SS among patients with RA was 30%.* *• The prevalence of SS increased with increasing RA disease duration.* *• Identifying specific clinical characteristics of patients with RA with SS, such as a greater incidence of extra-articular manifestations and comorbidities, may help clinicians to better characterize this patient population.*

**Key Points**

• *The overall prevalence of SS among patients with RA was 30%.*

*• The prevalence of SS increased with increasing RA disease duration.*

*• Identifying specific clinical characteristics of patients with RA with SS, such as a greater incidence of extra-articular manifestations and comorbidities, may help clinicians to better characterize this patient population.*

## Introduction

Sjögren’s syndrome (SS) is a systemic autoimmune disease with a wide variety of presentations which can be characterized as either glandular (e.g., dry eyes and/or mouth) or extraglandular (e.g., musculoskeletal, cutaneous, neurologic, pulmonary, or renal) manifestations [1, 2]. SS frequently occurs with another autoimmune disease such as rheumatoid arthritis (RA) [2, 3]; however, the prevalence and characterization of SS associated with RA are poorly understood [2, 4–6]. SS is commonly classified as “primary” or “secondary,” depending on whether or not a coexisting autoimmune condition is present. Recent publications, however, favor more descriptive terms, such as “SS alone” or “SS associated with systemic or organ-specific autoimmune diseases.” This trend has been developed to more accurately describe secondary SS in terms of which autoimmune disease is associated with SS and because the autoimmune conditions are coexisting rather than one being secondary to another [3].

A meta-analysis of 18 studies investigating SS associated with RA found a global prevalence of 19.5% [7]. Prevalence data for SS associated with RA in the USA are limited; one registry reported a rate of 10.3% [8]. Rate estimates from several European studies range from 7 to 31% [9–11]. The presence of SS adds to the RA disease burden, negatively impacts patients’ daily lives, and is associated with increased comorbidities, pain, fatigue, and joint damage, and a higher mortality rate [8, 12, 13].

SS may be a marker of more aggressive joint disease in patients with RA [8]; thus, identifying specific characteristics for patients with RA with SS may help clinicians to better characterize this population. The aims of this analysis were to assess the prevalence of SS associated with RA and to compare baseline characteristics of patients with RA with and without SS in the USA.

## Materials and methods

### Data source

The Corrona RA registry is an independent, prospective, national, observational cohort. Patients are recruited from 182 private and academic practice sites across 42 US states, with 781 participating rheumatologists. As of June 2019, the Corrona RA registry included information on 52,757 patients. Data on 397,236 patient visits and approximately 188,161 patient-years of follow-up time have been collected, with a mean (standard deviation [SD]) patient follow-up of 4.5 (3.8; median, 3.3) years.

### Study population

Patients aged ≥ 18 years with rheumatologist-diagnosed RA enrolled in the Corrona RA registry between 22 April 2010 and 28 February 2018 were included. SS status was determined from a yes/no variable based on physician diagnosis, reported into the Corrona RA registry at enrollment/follow-ups. For SS patients, the index date was defined as the date the provider first reported SS status as yes, and for patients without SS, it was the date on which the provider first reported SS status as no. Patients were included if they had ≥ 1 visit assessing the SS status (yes/no) and ≥ 12 months of follow-up after the index date to allow time for SS diagnosis. Patients with missing SS information were excluded. The presence of SS was determined by the clinical signs and symptoms of dry eyes, and/or dry mouth, unrelated to medications. Physician forms included a yes/no question regarding secondary SS status. Baseline was defined as the index visit in which a patient first reported a diagnosis of SS and RA or RA alone.

### Study outcomes

The primary outcome was the unadjusted prevalence of physician-reported SS associated with RA. Secondary outcomes included unadjusted prevalence of SS by RA disease duration and assessment of patients’ baseline characteristics by SS status.

### Statistical analysis

Unadjusted prevalence rate for SS associated with RA was calculated, using the proportion exact confidence intervals by Stata Software (version 15.1), excluding patients without 12-month follow-up after first SS report. For both prevalence of SS associated with RA and SS by RA disease duration, the 95% confidence intervals (CI) for the proportion of patients with SS alone and SS associated with RA were calculated based on a normal distribution. Baseline characteristics were summarized using descriptive statistics; frequency and percentage were calculated for categorical variables and means (SD) were calculated for continuous variables. Health status was measured by the five domains of the EuroQoL 5-dimension questionnaire: mobility, self-care, usual activities, pain/discomfort, and anxiety/depression [14]. All analyses were performed using Stata Release 15 (StataCorp LLC, College Station, TX, USA).

## Results

### Patient disposition

**Fig. 1:**
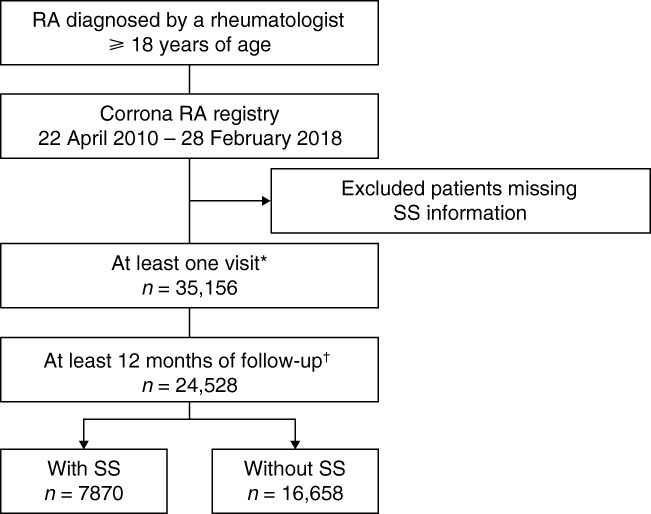
Patient disposition. Asterisk indicates yes/no to having SS. Dagger mark indicates after the first capture of SS data in patients with a diagnosis of no SS. RA = rheumatoid arthritis; SS = Sjögren’s syndrome

Data were available for 35,156 patients with RA who had a known SS status, of whom 24,528 patients had at least 12 months of follow-up (Fig. 1). Approximately one-third of patients had RA with SS (*n* = 7870; 32.1%).

### Prevalence of SS in patients with RA

**Fig. 2:**
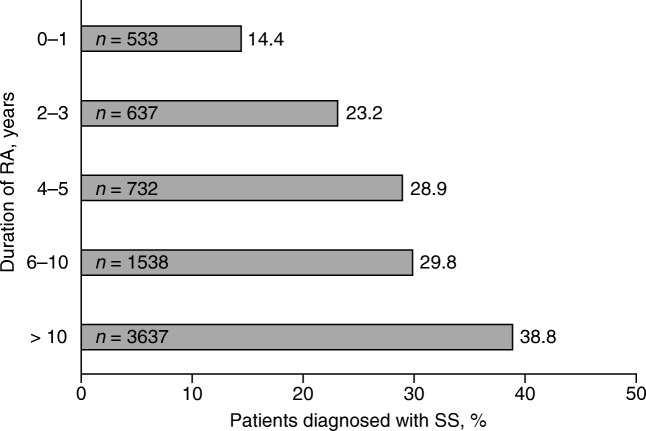
Unadjusted prevalence of SS in patients with RA by duration of disease. Asterisk indicates patients included in analysis = 7077. RA = rheumatoid arthritis; SS = Sjögren’s syndrome

The unadjusted rate (excluding patients with < 12 months of follow-up) for the prevalence of SS in patients with RA was 30.0% (95% CI 29.4%, 30.6%). The unadjusted SS rate increased with RA disease duration from 14.4% in patients with disease duration of 0 to 1 years to 38.8% in patients with disease duration of > 10 years (Fig. 2).

### Clinical characteristics

**Fig. 3:**
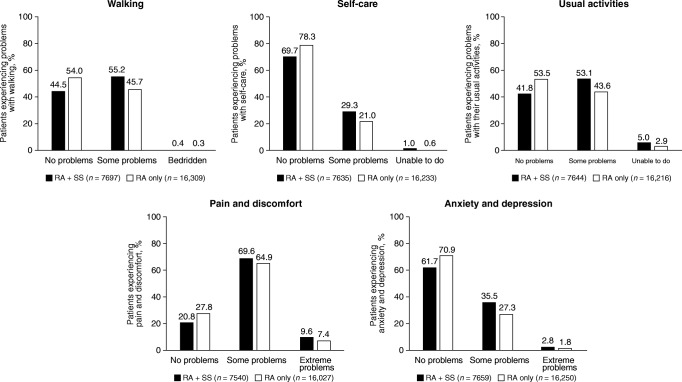
Health status as measured by the five domains of the EuroQoL 5-dimension questionnaire: mobility, self-care, usual activities, pain/discomfort, and anxiety/depression. Health status was measured at the index date. RA = rheumatoid arthritis; SS = Sjögren’s syndrome

Compared with patients with RA only, patients with RA with SS were more likely to be female, older and have longer RA disease duration (Table 1). There were fewer patients with RA with SS in full-time employment and more disabled or retired patients compared with patients with RA only. Patients with RA with SS had more baseline comorbidities (hypertension, cardiovascular disease, and malignancies), erosive disease, and subcutaneous nodules than patients with RA only. Patients with RA with SS experienced twice the incidence of serious infections requiring hospitalization or intravenous treatment than patients with RA only. Patients with RA with SS, versus RA only, were more likely to be seropositive (anti-cyclic citrullinated peptide-positive, rheumatoid factor-positive, and double-positive) and have a higher mean clinical disease activity index score (Table 1). Patients with RA with SS were more likely to be taking abatacept or other non-tumor necrosis factor inhibitor biologic/targeted synthetic (ts) disease-modifying antirheumatic drugs (DMARDs) than patients with RA only. Additionally, they were more likely to have previously used > 1 conventional synthetic DMARD and biologic/tsDMARD (Table 1). Compared with patients with RA only, patients with RA with SS were more likely to have experienced morning stiffness and have higher mean modified health assessment questionnaire [15], patient pain, and Patient Global Assessment scores. Patients with RA with SS were more likely to report difficulties with walking, self-care, usual activities, pain and discomfort, and anxiety and depression than patients with RA only (Fig. 3).

**Table 1:** Patient demographic and clinical characteristics at index date

	Patients with RA with SS (*n* = 7870)	Patients with RA only (*n* = 16,658)
Age, years, mean (SD)	62.5 (11.9)	59.2 (13.1)
Sex, female	6617 (84.4)	12,229 (73.8)
Duration of RA, years, mean (SD)	13.6 (11.0)	9.5 (9.2)
Work status	*n* = 7692	*n* = 16,373
Full-time	2142 (27.8)	6530 (39.9)
Part-time	607 (7.9)	1389 (8.5)
Disabled	1237 (16.1)	1716 (10.5)
Retired	2986 (38.8)	5169 (31.6)
Other	720 (9.4)	1569 (9.6)
Comorbidities		
Hypertension	2909 (37.0)	5214 (31.3)
CV disease*	1219 (15.5)	1710 (10.3)
Malignancy^†^	1223 (15.5)	1821 (10.9)
Serious infections^‡^	795 (10.1)	845 (5.1)
Diabetes	775 (9.8)	1416 (8.5)
Asthma	403 (5.1)	590 (3.5)
COPD	270 (3.4)	355 (2.1)
ILD/pulmonary fibrosis	81 (1.0)	81 (0.5)
Anti-CCP-positive, n/m (%)	1999/3420 (58.5)	4076/7451 (54.7)
RF+, n/m (%)	2983/4296 (69.4)	6338/9492 (66.8)
Erosive disease, n/m (%)	2480/6650 (37.3)	4230/12,406 (34.1)
Subcutaneous nodules, n/m (%)	2700/7869 (34.3)	2886/16,640 (17.3)
CDAI, mean (SD)	13.4 (12.8)	11.3 (11.9)
Current medication use		
TNFi biologic	2924 (37.2)	6390 (38.4)
Abatacept	591 (7.5)	980 (5.9)
Other non-TNFi biologic/tsDMARD	781 (9.9)	962 (5.8)
csDMARD	7227 (91.8)	15,650 (93.9)
Number of prior biologics/tsDMARDs		
0	2583 (32.8)	7593 (45.6)
1	2656 (33.7)	5592 (33.6)
≥ 2	2631 (33.4)	3473 (20.8)
Number of prior csDMARDs		
0	367 (4.7)	1704 (10.2)
1	2984 (37.9)	8016 (48.1)
≥ 2	4519 (57.4)	6938 (41.6)
Patient-reported outcomes		
mHAQ score, mean (SD); *n*	0.4 (0.5); 7659	0.3 (0.4); 16,466
Pain score, mean (SD); *n*	37.2 (28.7); 7829	31.2 (27.5); 16,549
Global assessment score, mean (SD); *n*	35.3 (27.3); 7830	28.9 (26.4); 16,549
Morning stiffness, n/m (%)	5884/7717 (76.2)	11,628/16,334 (71.2)

Data are *n* (%) unless otherwise stated

*Anti-CCP*, anti-cyclic citrullinated peptide; *CDAI*, Clinical Disease Activity Index; *COPD*, chronic obstructive pulmonary disease; *csDMARD*, conventional synthetic disease-modifying antirheumatic drug; *CV*, cardiovascular; *ILD*, interstitial lung disease; *mHAQ*, modified health assessment questionnaire; *n/m*, number of patients by total number of patients in the analysis; *RA*, rheumatoid arthritis; *RF*, rheumatoid factor; *SD*, standard deviation; *SS*, Sjögren’s syndrome; *TNFi*, tumor necrosis factor inhibitor; *tsDMARD*, targeted synthetic disease-modifying antirheumatic drug

*History of coronary artery disease, myocardial infarction, congestive heart failure requiring hospitalization, acute coronary syndrome, unstable angina, cardiac revascularization procedure, cardiac arrest, ventricular arrhythmia, stroke, transient ischemic attack, or other CV events

^†^History of lung cancer, breast cancer, lymphoma, skin cancer (melanoma and squamous), or other cancer

^‡^Infection required hospitalization or IV treatment

## Discussion

In the large US-based Corrona RA registry, the prevalence of RA with SS was 30%, which is higher than that previously reported in the US (10.3%) [8] and toward the higher end of the range reported for Europe (7 to 31%) [9–11]. The variance in the rates may be due to many reasons, including sample size, the SS classification criteria used, study population differences, and patient—versus physician—reported diagnoses.

Conflicting results have been published regarding whether RA disease duration affects the prevalence of SS, although several studies had low patient numbers [4, 9, 11, 16–18]. An analysis of the Oslo Rheumatoid Arthritis Registry (ORAR; *n* = 631) found no correlation between SS symptoms and RA disease duration [11]; whereas a cross-sectional study in an Indian hospital (*N* = 199) showed that patients with secondary SS had a longer disease duration than those without secondary SS [18]. In addition, a larger study in Spanish patients (*n* = 788) diagnosed by rheumatologists found that the prevalence of SS increased with RA disease duration from an incidence (95% CI) of 2.3 (1.4, 3.7) at 2 years to 9.1 (6.9, 11.8) at 10 years [9]. This finding is supported by our study, which to the best of our knowledge is the largest to date investigating patients with RA with SS (*n* = 7870), and showed that a higher prevalence of RA with SS was observed as RA duration increased.

The comparison of the baseline characteristics showed that compared with patients with RA only, patients with RA with SS were older, more likely female, and had longer RA disease duration. In addition, RA with SS was associated with seropositivity; more severe RA; more health-related difficulties such as pain and anxiety; a lower level of employment; and a greater incidence of other extra-articular manifestations and comorbidities. There are limited data available in the literature regarding the characteristics of patients with RA and SS [8]; however, the data reported here are supported by a smaller cross-sectional study of 85 patients with RA which showed that patients with RA with SS were more likely to be female and have longer disease duration and higher frequency of RF and/or anti-citrullinated protein antibody (ACPA) positivity [8]. Similarly, a single-center study (*n* = 74) found that higher disease scores and frequencies of RF positivity were reported in patients with RA with SS compared with those with either RA or SS alone [4].

In contrast, a small cross-sectional study found no differences between patients with RA with SS (*n* = 11) and those with RA alone (*n* = 296) regarding age, sex, RA disease duration, disease activity score, and ACPA seropositivity; however, it showed that patients with RA and secondary SS had a tendency for higher numbers of tender and swollen joints and pain [17]. Data from a Finnish Registry showed that patients with secondary SS (*n* = 709) were more likely to have hematologic malignancies than those with RA alone (*n* = 9469; standard incidence ratio, 3.1% vs 1.7%) [19]. The differences between groups in these descriptive studies may be a result of the low number of patients with RA with SS in the former study. The low patient numbers make comparisons between studies difficult as differences may be due to SS, factors associated with SS, or the randomness of the data.

A strength of our study is the source of our patient data. Corrona is the largest US disease-registry that collects data directly from both providers and patients at the time of a routine clinical encounter, which allowed for a very large number of patients with RA and SS to be investigated. One potential limitation in every registry-based analysis is whether findings are representative of, and generalizable to, the broader population. Corrona does include rheumatology practices participating throughout the country in rural and urban areas in academic and private settings and with access to broad geographic locations and patients with diverse sociodemographic origins. In addition, prior work compared Medicare patients enrolled in Corrona with those who are not part of the registry and found similar demographic and comorbidity characteristics, supporting the generalizability of the Corrona registry [20]. As our study used a physician-reported diagnosis of SS associated with RA, it is possible that providers may differ in the criteria used to diagnose patients. Differences in diagnosis criteria are shown throughout the literature, suggesting a need for standardized diagnosis criteria for patients with SS and RA which are easy to implement in everyday practice. In addition, patients in the RA alone group required a questionnaire entry for SS (yes/no), potentially leading to under ascertainment.

## Conclusions

In this large, US patient population, patients with RA with SS had a higher disease burden than those with RA alone; a higher prevalence of SS was observed as RA disease duration increased. Identifying specific characteristics of patients with RA with SS may help clinicians to better understand this patient population and the extra-articular manifestations of RA. Additional studies are warranted to further understand the full burden of SS in patients with RA and its impact on clinical and patient-reported outcomes.

## Data Availability

The datasets generated during and/or analyzed during the current study are available from the corresponding author on reasonable request.
